# Stabilisation of Laryngeal AL Amyloidosis with Long Term Curcumin Therapy

**DOI:** 10.1155/2015/910528

**Published:** 2015-06-25

**Authors:** Terry Golombick, Terrence H. Diamond, Arumugam Manoharan, Rajeev Ramakrishna

**Affiliations:** ^1^Department of Endocrinology, St George Hospital, Sydney, NSW 2217, Australia; ^2^Southern Sydney Haematology, University of Wollongong, NSW 2500, Australia

## Abstract

Multiple myeloma (MM), smoldering myeloma (SMM), and monoclonal gammopathy of undetermined significance (MGUS) represent a spectrum of plasma cell dyscrasias (PCDs). Immunoglobulin light chain amyloidosis (AL) falls within the spectrum of these diseases and has a mortality rate of more than 80% within 2 years of diagnosis. Curcumin, derived from turmeric, has been shown to have a clinical benefit in some patients with PCDs. In addition to a clinical benefit in these patients, curcumin has been found to have a strong affinity for fibrillar amyloid proteins. We thus administered curcumin to a patient with laryngeal amyloidosis and smoldering myeloma and found that the patient has shown a lack of progression of his disease for a period of five years. This is in keeping with our previous findings of clinical benefits of curcumin in patients with plasma cell dyscrasias. We recommend further evaluation of curcumin in patients with primary AL amyloidosis.

## 1. Introduction

Immunoglobulin light chain amyloidosis (AL) along with multiple myeloma (MM), smoldering myeloma (SMM), and monoclonal gammopathy of undetermined significance (MGUS) represent a spectrum of plasma cell dyscrasias (PCDs) [1]. AL is a rare and serious disorder characterized by the deposition of amyloid fibrils in different tissues with a mortality rate of more than 80% within 2 years of diagnosis, particularly if associated with renal or cardiac involvement [2, 3]. AL may coexist with any of the PCDs. It has been reported that up to 30% of MM patients may have subclinical amyloid deposits. In the case of SMM, no treatment will be directed against the plasma cell clone, potentially allowing for unbridled progression of the AL. The subtype of amyloidosis called AL is the most heterogeneous form of the disease, consistent with the fibril protein being unique in each patient. Virtually any organ system excluding the brain can be affected and in almost any combination.

Curcuma longa or turmeric is a tropical plant native to southern and southeastern tropical Asia. It is a perennial herb belonging to the ginger family. The most active component in turmeric is curcumin [4]. Numerous reports suggest that curcumin has chemopreventive and chemotherapeutic effects. Curcumin has been shown to inhibit the proliferation of a wide variety of tumor cells, including multiple myeloma cells through the downregulation of IL-6 and NF-*κ*B [5]. Studies by Golombick et al. [6–9] have found that curcumin decreases paraprotein load, bone turnover, free light chains, and % plasma cells in the bone marrow of some MGUS and smoldering myeloma patients. In addition to the beneficial effects noted above, it has been suggested that curcumin may have beneficial effects on diseases characterized by the formation of aggregated fibrillar protein deposits [10]. Curcumin has strong affinity for fibrillar amyloid proteins and is already used to stain* in vitro* tissue sections from individuals affected with neurodegenerative disease such as Alzheimer's and Parkinson's disease [11].

Based on the demonstratable effects of curcumin in MGUS and SMM, we administered curcumin to a patient with IgG lambda smoldering myeloma with supraglottic AL amyloidosis. In this case report we demonstrate the beneficial effects of curcumin on the size of his laryngeal amyloid deposit after five years of therapy.

## 2. Case Presentation

**Figure 1:**
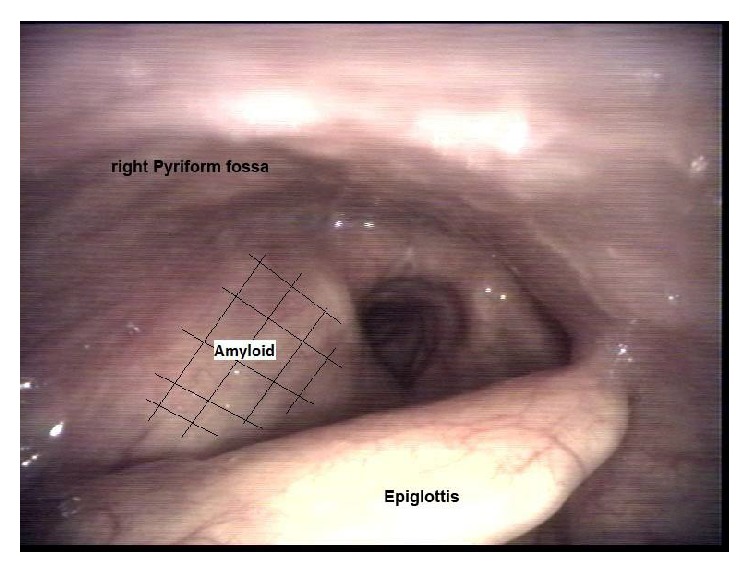
Supraglottic amyloid.

**Figure 2:**
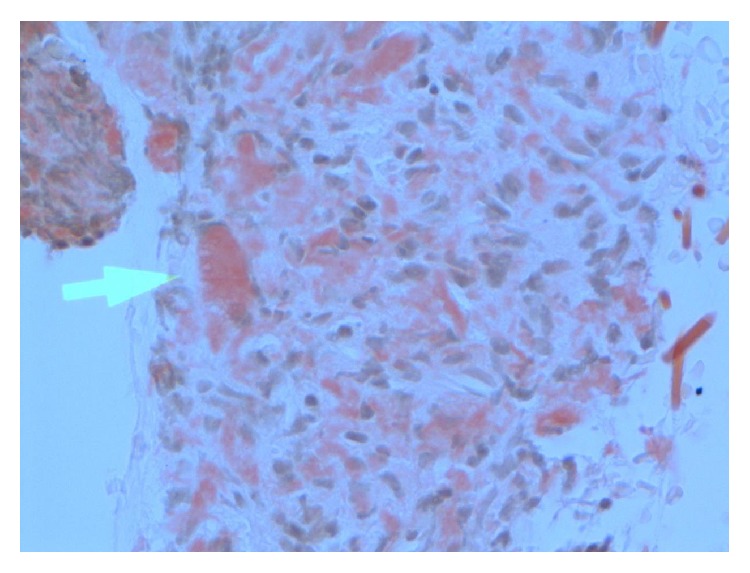
Amyloid tumour of the larynx. X 40—connective tissue partly covered by squamous mucosa. Within the connective tissue is eosinophilic material which stains positively with Congo red. This Congo red stained material shows apple green birefringence under polarised light. The appearances are consistent with amyloid.

A 72-year-old male patient presented to the Haematology Clinic in 2006 for evaluation of laryngeal amyloidosis, secondary to smoldering myeloma. This was incidentally discovered by Doppler studies after being investigated for a cerebrovascular event. He denied symptoms of dysphagia or dysphonia. His comorbidities included spinal canal stenosis due to osteoarthritis, diet controlled diabetes, and hypertension. He had no history of fever, weight loss, recurrent infections, or skeletal events. Direct laryngoscopy confirmed an expansion of the supraglottis without evidence of soft tissue invasion or fixation to the prevertebral tissue. This involved mostly the region of the aryepiglottic fold and extended down to the false vocal fold on the right side causing effacement of the right pyriform fossa (Figure 1). It was submucosal without ulceration. Vocal cord mobility was normal and there was no evidence of lymphadenopathy. Biopsies confirmed the diagnosis of amyloidosis, as an AL type (Figure 2). A diagnosis of SMM was established by bone marrow dyscrasia (18% plasma cell infiltration, IgG lambda) and an elevated plasma paraprotein (14 g/L) but a negative 24-hour urine Bence Jones protein. The B2 microglobulin (2.2 mg/L), EUC, and calcium levels were normal. A skeletal survey, and chest, abdomen, and pelvic CT scan excluded lytic and soft tissue lesions. Antimyeloma therapy was not initiated as he was otherwise asymptomatic.

In 2008, repeat laryngoscopy demonstrated progressive disease. There was a significant increase in the prominence of the right thyroid cartilage and supraglottic swelling. The plasma paraproteinemia (14 g/L) and free light chain ratio remained stable and the 24-hour urine Bence Jones Protein remained negative. A repeat bone marrow biopsy revealed persistent plasmacytosis (18%) and negative Congo red stains for amyloidosis. In June 2008 the patient had three courses of melphalan (8 mg daily for 4 days) and dexamethasone (12 mg daily for 4 days) chemotherapy which resulted in an improvement in his paraproteinemia (6 g/L). He declined further chemotherapy due to dexamethasone-related weight gain and secondary diabetes and melphalan-related gastrointestinal side effects and cytopenias. In 2009, he developed worsening in back pain and was found to have CT evidence of a sacral lytic lesion. He was treated with localised external beam radiation therapy for pain control but with no systemic therapy.

In 2009, the patient commenced curcumin therapy at a dose of 1500 mg per day, gradually increasing to 3600 mg per day. By October 2010, the supraglottic swelling had decreased in size. In March and October 2011, the patient underwent video assessment of the larynx and no progressive disease was evident. The patient has remained extremely well apart from chronic arthritic pain. MRI scans excluded new or progressive skeletal disease, particularly his sacrum. Serial haematological and otolaryngeal investigation, performed annually from 2010 to 2014, has demonstrated stable disease. The biomarkers performed over a 5-year period on curcumin therapy are outlined in Table 1.

## 3. Discussion

The amyloidoses are a group of diseases that have in common the extracellular deposition of pathologic, insoluble fibrils in various tissues and organs. The fibrils have a characteristic *β*-pleated sheet configuration. Many different proteins can form amyloid fibrils, and the types of amyloidosis are classified on the basis of the amyloidogenic protein as well as by the distribution of amyloid deposits being either systemic or localized [12]. In the systemic form, the amyloidogenic protein is produced at a site that is distant from the site of deposition. In contrast, in localized disease, the amyloidogenic protein is produced at the site of deposition.

Light chain (AL) amyloidosis is the most common type of systemic amyloidosis and appears to be more common than previously thought [2, 13]. The amyloidogenic protein in AL amyloidosis is an Ig light chain or a fragment of a light chain that is produced by a clonal population of plasma cells in the bone marrow. Plasma cell burden in this disorder varies and is typically 5 to 10% [3] and in approximately 10 to 15% of patients, AL amyloidosis occurs in association with multiple myeloma [14]. It appears that both the light chain variable region (Ig*V*_*L*_) germ line genes used by AL clones and the plasma cell burden influence AL organ tropism (selection of the site of deposition) [15].

The treatment of AL amyloidosis involves the same chemotherapeutic agents used to treat multiple myeloma. The goal is to reduce or stop the production of monoclonal light chains by reducing or eliminating clonal plasma cells [16]. Once the process of active deposition is halted, amyloid deposits are slowly resorbed by the body. Therapy that lowers the amyloidogenic FLC concentration can stop accumulation of AL amyloid deposits, lead to their regression, and improve survival. Treatment has been difficult and unsatisfactory and has hitherto involved intensive chemotherapy [17]. Several studies have evaluated the use of the immunomodulatory agents (thalidomide, bortezomib, or lenalidomide), in combination with melphalan or cyclophosphamide and/or dexamethasone in patients with AL amyloidosis [18–20]. Venner et al. [18] examined upfront therapy with cyclophosphamide, bortezomib, and dexamethasone (CVD) versus cyclophosphamide, thalidomide, and dexamethasone (CTD) and showed that the overall response rates were 71.0% versus 79.7% in the CVD and CTD arms, respectively, (*P* = 0.32) [18]. One-year overall survival (OS) was 65.2% and 66.7% for CVD and CTD, respectively (*P* = 0.87). The median progression-free survival (PFS) was 28.0 m and 14.0 m for CVD and CTD, respectively (*P* = 0.039). According to outcomes performed at 6 months, the CR rate with CVD was 59.6% versus 34.0% for CTD (*P* = 0.03). Both regimens were unable to overcome the high rate of early deaths in AL amyloidosis. A phase II trial of lenalidomide and dexamethasone in the treatment of AL amyloidosis has shown an overall haematological response rate of 67% [20]. Mahmood et al. [21] from the National Amyloidosis Centre in the UK evaluated lenalidomide and dexamethasone therapy for systemic AL amyloidosis following prior treatment with thalidomide or bortezomib regimens. The overall haematological response rate was similar at 61%. These authors also noted that long term therapy achieves a marked improvement in organ responses and suggested that immunomodulatory effects of lenalidomide therapy might enhance the otherwise slow natural regression of amyloid deposits.

A recent* in vitro* study conducted in our laboratory [22] found that curcumin enhances the cytotoxic effects of lenalidomide in human multiple myeloma cells via suppression of the cereblon gene. Our findings suggest that curcumin can not only potentiate the cytotoxic effect of lenalidomide but also enhance the chemosensitizing effects of this agent. Considering the similarity in the mechanisms of action of lenalidomide and curcumin, it is possible that the stabilisation of the amyloid deposit in this patient was due to the long term effects of curcumin therapy.

Patients with myeloma and associated amyloid deposition tend to progress and it is unusual for the disease to remain indolent without chemotherapy. Our patient received only 3 months of low dose oral chemotherapy and radiation therapy for a sacral lytic lesion and has been maintained exclusively on curcumin therapy. The lack of progression of his laryngeal amyloidosis and smoldering myeloma over a 5-year period is in keeping with our previous findings of clinical benefits of curcumin in patients with plasma cell dyscrasias [6–9]. We recommend further evaluation of curcumin in patients with primary AL amyloidosis.

## Figures and Tables

**Table 1:** Haematological and biochemical data performed at first visit (February 2006) and at 3 months (2009) after commencing curcumin therapy and thereafter annually (2010–2014).

Variable	2006	2009	2010^*∗*^	March 2013	March 2014
Bone marrow biopsy (% plasma cells)	18	2	5	<2	<2
Paraprotein (g/L)	14	6	1	Trace	Trace
Free light chain ratio (0.3–1.7)		1.7	1.3	0.81	0.68
Lambda FLC (<26.3 mg/L)		9	4	37	40
Kappa FLC (<19.4 mg/L)		15	5	30	27
Globulin (22–38 g/L)		29	23	30	30
Calcium (mmol/L)		2.32	2.34	2.46	
Ig G (6.2–14.4 g/L)	20	10.01	9.5	9.5	9.4
Ig M (0.48–3.04 g/L)		0.71	0.85	0.87	0.95
Ig A (0.6–3.96 g/L)		1.63	1.68	1.42	1.35
Haemoglobin (128–175 g/L)	157	120	139	135	136
WCC (4.0–11.0 × 10^9^/L)	6	3.2	4.3	6.8	6.1
Platelets (150–450 × 10^9^/L)	236	114	164	171	174
LDH (u/L)	167	163	133	142	140
B2 microglobulin (g/L)	2.2	2.5	3.1	3.4	3.7
Serum creatinine (60–120 *μ*mol/L)		109	135	127	117
eGFR (>89 mL/min)		57	45	46	51
u-creat (8.8–17.6 mmol/L)		10.3	8.1	6.6	
u-prot (0.01–0.20 mg/day)		0.15	0.06	0.05	0.07

^*∗*^After 3 months of curcumin therapy.

“u-creat” refers to urinary creatinine and “u-prot” refers to urinary-protein.
